# P-2107. Invasive fungal sinusitis in patients with hematological malignancies: a 20-year study from a tertiary academic US hospital system

**DOI:** 10.1093/ofid/ofaf695.2271

**Published:** 2026-01-11

**Authors:** Tejas S Athni, Carolyn Strauch, Muneerah Aleissa, Simran Gupta, Alexis Liakos, Alexandra Tong, Inia Andrea Perez Villa, Esther Arbona Haddad, Victor Kovac, Rahul S Vedula, Alice Z Maxfield, Regan Bergmark, Amy C Sherman

**Affiliations:** Harvard Medical School, Boston, MA; BIDMC, Boston, Massachusetts; Princess Nourah University, Riyadh, Massachusetts; Brigham and Women's Hospital, Chestnut Hill, Massachusetts; Brigham & Women's Hospital/Dana-Farber Cancer Institute, boston, Massachusetts; Brigham and Women's Hospital, Chestnut Hill, Massachusetts; Brigham and Women's Hospital, Chestnut Hill, Massachusetts; Brigham and Women's Hospital, Chestnut Hill, Massachusetts; Brigham and Women's Hospital, Chestnut Hill, Massachusetts; Dana Farber Cancer Institute, Boston, MA; Harvard Medical School, Boston, MA; Harvard Medical School, Boston, MA; Brigham and Women's Hospital, Chestnut Hill, Massachusetts

## Abstract

**Background:**

Invasive fungal sinusitis (IFS) can profoundly impact individuals with hematological malignancies, with high mortality rates, rapid disease progression, and slow response to medical therapy. With an evolving therapeutic landscape, outcomes for patients with heme malignancies and IFS warrant further evaluation.

**Figure:**
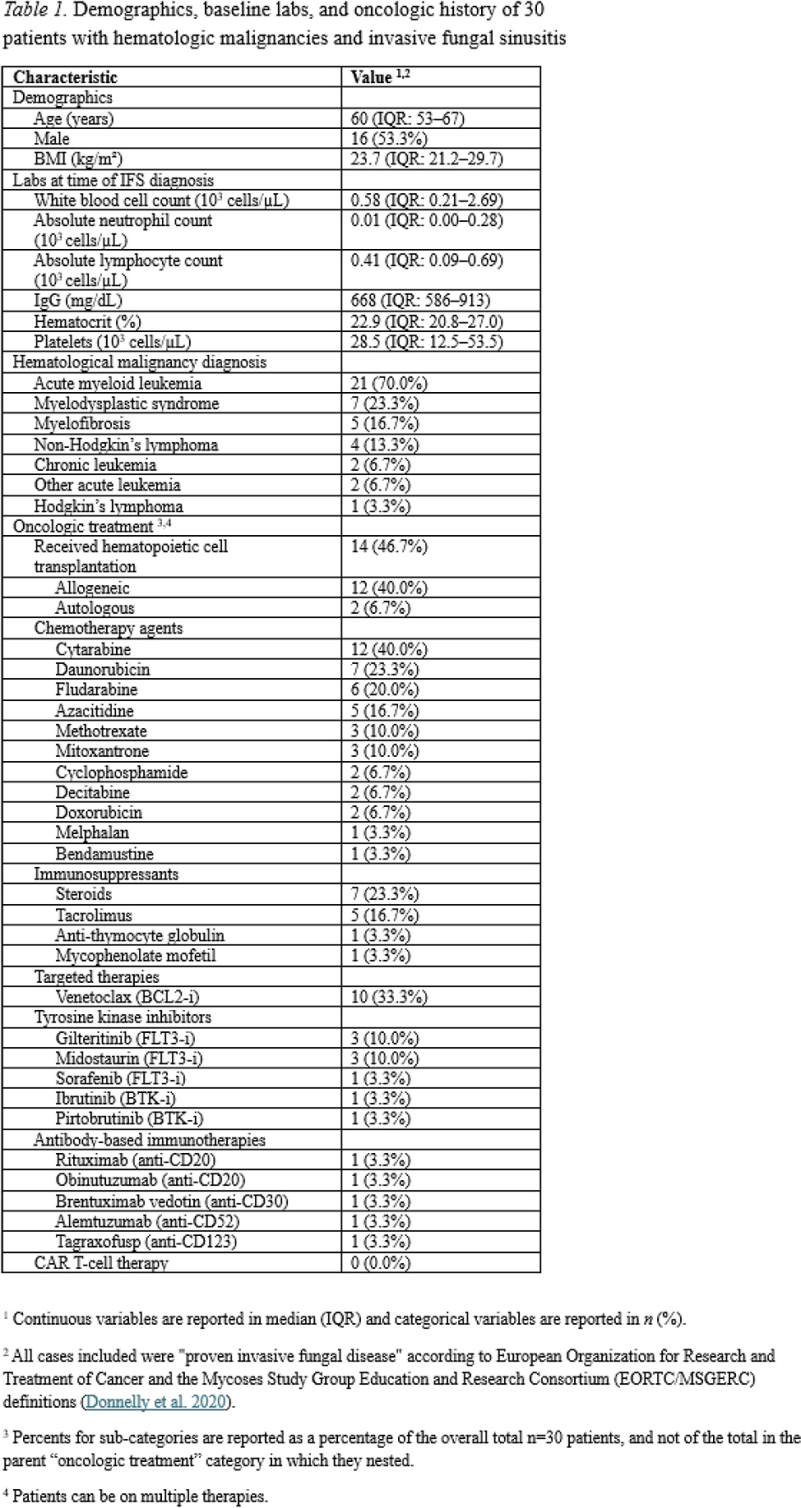

**Figure:**
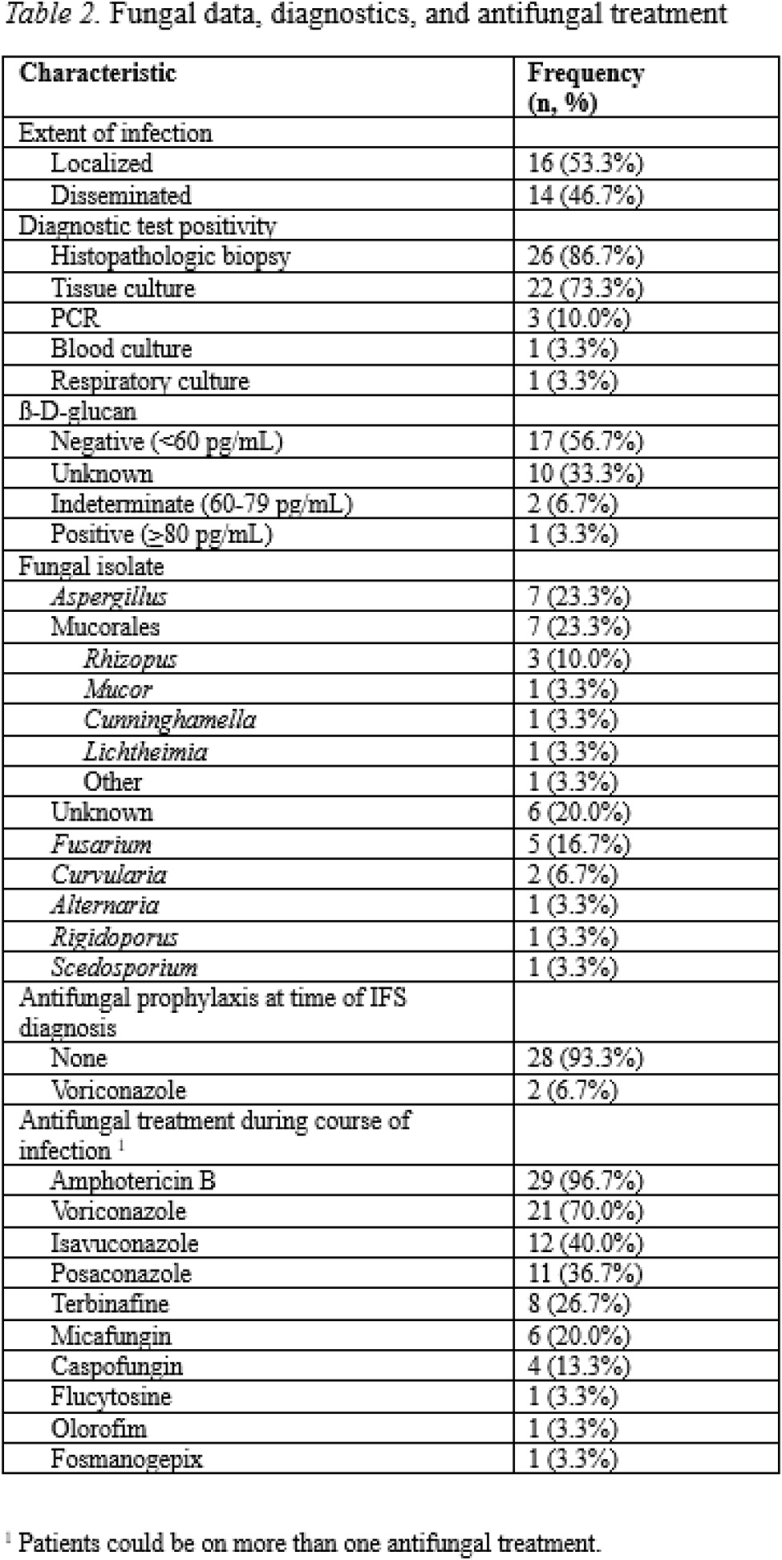

**Methods:**

We performed a single-center, retrospective review of patients with hematologic malignancies who developed proven IFS. We characterized patient demographics; oncologic history; mycologic, radiographic, and surgical data; sinonasal endoscopy findings; and mortality outcomes. Frequencies were reported for categorical variables. Medians and interquartile ranges were reported for continuous variables (R v4.3.1).

**Figure:**
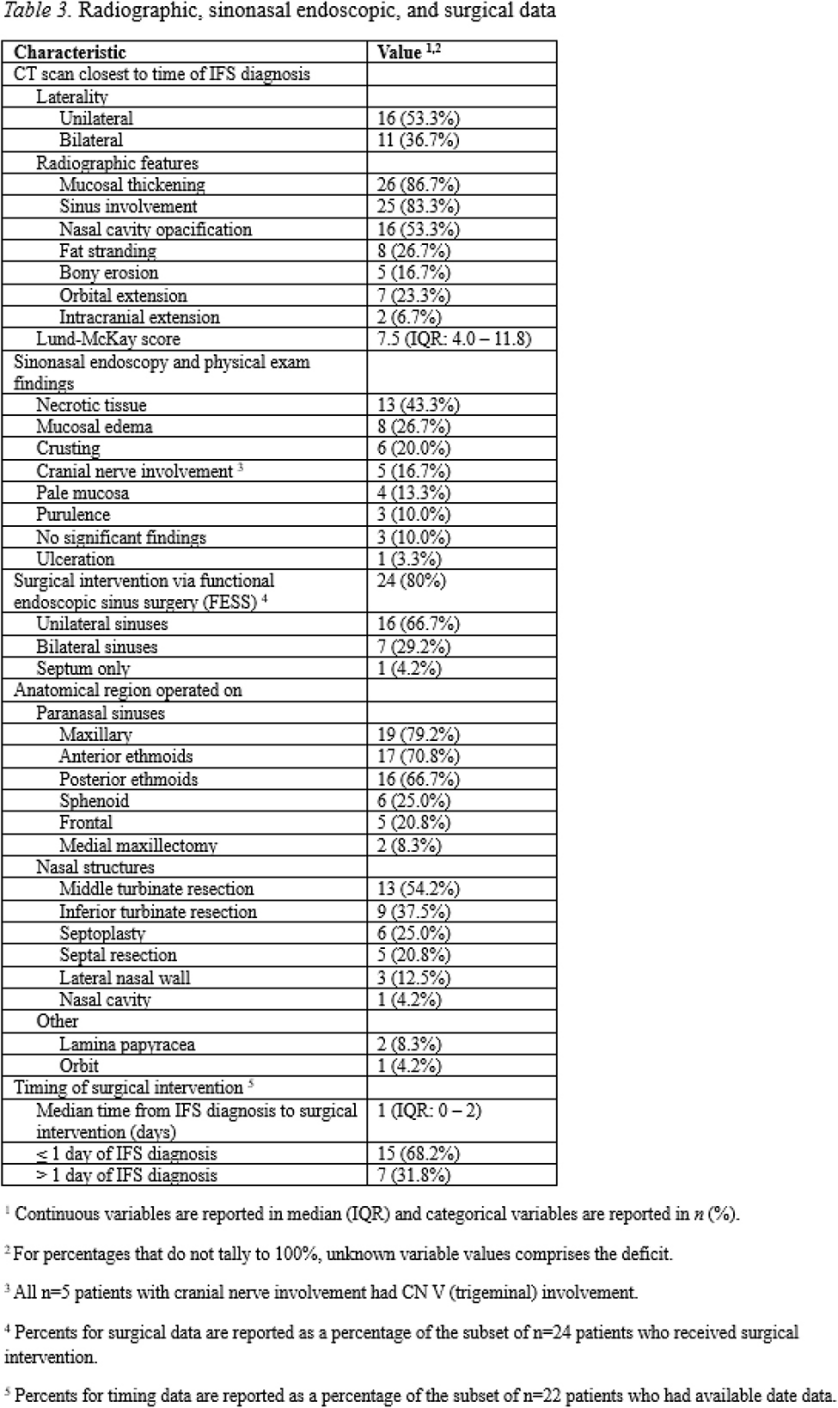

**Figure:**
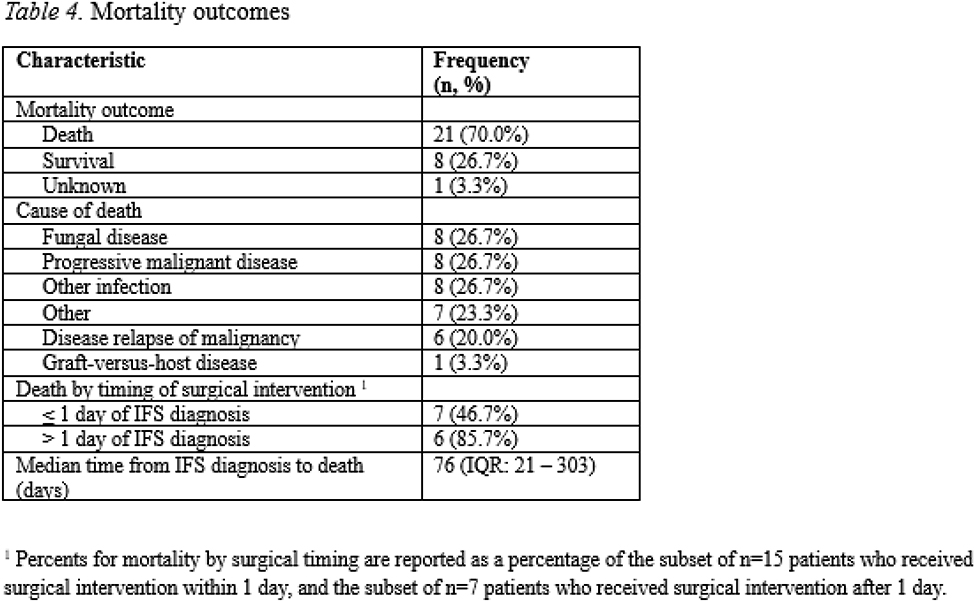

**Results:**

We identified 30 patients with confirmed IFS diagnosed between 2005-2024, with characteristics described in Table 1. Most patients had a diagnosis of acute myeloid leukemia (70.0%). Within 6 months prior to IFS, patients had received cytarabine (40.0%) and steroids (23.3%), with newer agents including venetoclax (33.3%), tyrosine kinase inhibitors (26.7%), brentuximab vedotin (3.3%), and tagraxofusp (3.3%). Mycologically 53% of patients had localized and 47% had disseminated fungal infection (Table 2). Histopathologic biopsy was positive in 86.7%, tissue culture in 73.3%, and PCR in 10.0% of patients. The most common fungal isolates were *Aspergillus* (23.3%), Mucorales (23.3%), and *Fusarium* (16.7%). Most patients had not received antifungal prophylaxis (93.3%). Amphotericin B (96.7%) and voriconazole (70.0%) were the most common initial treatments. Novel therapies included olorofim (3.3%) and fosmanogepix (3.3%). Half of cases were unilateral (53.3%), with further anatomical and surgical characteristics described in Table 3. Median time from IFS diagnosis to surgery was 1 day (IQR: 0-2). Overall mortality was 70.0%, with 26.7% due to IFS and median time from IFS diagnosis to death of 76 days (IQR: 21-303) (Table 4). Surgery within 1d of IFS diagnosis had 46.7% mortality, while surgery after 1d had 85.7% mortality.

**Conclusion:**

Despite novel therapies and aggressive interventions, patients with IFS had high mortality. We seek to further determine specific prognostic factors that portend positive outcomes for patients with IFS.

**Disclosures:**

Rahul S. Vedula, MD, NA: inventor on patent (PCT/US2020/049257) Regan Bergmark, MD MPH, Analysis Group: Advisor/Consultant|i-Mab Biopharma: Advisor/Consultant

